# CSF Aβ_1–42_ level is associated with cognitive decline in early Parkinson’s disease with rapid eye movement sleep behavior disorder

**DOI:** 10.1186/s40035-018-0129-5

**Published:** 2018-10-08

**Authors:** Maowen Ba, Guoping Yu, Min Kong, Hui Liang, Ling Yu

**Affiliations:** 1 Department of Neurology, the Affiliated Yantai Yuhuangding Hospital of Qingdao University, Yantai City, Shandong 264000 People’s Republic of China; 2 Department of Neurology, Yantaishan Hospital, Yantai City, Shandong 264000 People’s Republic of China

**Keywords:** Parkinson’s disease, Rapid eye movement sleep behavior disorder, Cognitive decline, β-Amyloid

## Abstract

**Background:**

Rapid eye movement sleep behavior disorder (RBD) is associated with cognitive decline in early Parkinson’s disease (PD). However, the underlyling basis for this association remains unclear.

**Methods:**

Parkinson’s Progression Marker’s Initiative (PPMI) subjects underwent baseline RBD testing with RBD sleep questionnaire (RBDSQ). Serial assessments included measures of motor symptoms, non-motor symptoms (NMS), neuropsychological assessment, blood and cerebrospinal fluid (CSF) biomarkers. Up to three years follow-up data were included. We stratified early PD subjects into PD with RBD (RBDSQ score > 5) and PD without RBD groups. Then, we evaluated baseline biomarkers in each group as a predictor of cognitive decline using Montreal Cognitive Assessment (MoCA) score changes over three years in regression models.

**Results:**

Four hundred twenty-three PD subjects were enrolled at baseline, and a total of 350 PD subjects had completed 3 years of study follow-up with completely serial assessments. We found that at baseline, only CSF β-amyloid 1–42 (Aβ_1–42_) was significantly lower in PD subjects with RBD. On three years follow-up analysis, PD subjects with RBD were more likely to develop incident mild cognitive impairment (MCI) and presented greater cognitive decline in MoCA score. Lower baseline CSF Aβ_1–42_ predicted cognitive decline over 3 years only in PD subjects with RBD (β = − 0.03, *P* = 0.003). A significant interaction between Aβ_1–42_ and the 2 groups confirmed that this effect was indeed higher in PD with RBD than the other individual (β = − 2.85, *P* = 0.014).

**Conclusion:**

These findings indicate that CSF Aβ_1–42_ level is associated with global cognitive decline in early PD with RBD. The addition of CSF Aβ_1–42_ to RBD testing increase the likelihood of identifying those at high risk for cognitive decline in early PD.

## Background

Parkinson’s disease (PD) is one of age-related neurodegenerative diseases with a wide range of motor and nonmotor symptoms (NMS) [1, 2]. Rapid eye movement sleep behavior disorder (RBD) and cognitive impairement are two common NMS manifestations in PD [3–6]. The presence of RBD can predict a greater cognitive decline and a greater risk of developing dementia in advanced PD [7–10]. The relationship between RBD and cognitive decline has also been reported in a preliminary analysis of early PD subjects from the Parkinson’s Progression Markers Initiative (PPMI) cohort [11, 12]. However, no prior work has identified the underlyling basis of cognitive impairement in early PD with or without RBD.

Recent studies have suggested that several biomarkers including blood apolipoprotein E ɛ4 (ApoE4) genotype, cerebrospinal fluid (CSF) Aβ_1–42_ (lower) and tau (higher) have been associated with cognitive impairment in PD in cross sectional studies [13–16]. However, the results did not clarify the relationship between these biomarkers and RBD for cognitive decline in early PD. It is interesting to explore whether the presence of any of these biomarkers increases the risk of developing cognitive decline in early PD with RBD. So, the aims of this study were to (i) confirm the association between RBD and global cognitive decline in early PD over 3 years follow-up period, and (ii) further assess which biomarker is associated with cognitive decline in early PD with RBD. The PPMI therefore provides an early opportunity to explore relationships between cognitive findings and potentially influencing factors at the early stage of PD in a large, untreated, imaging-confirmed cohort of newly diagnosed PD patients, where medications do not confound motor and NMS measures.

## Methods

### Study samples

Data used in the preparation of this study were obtained from PPMI database (http://www.ppmi-info.org). The PPMI was a prospective cohort study of PD patients untreated at enrollment and sponsored by The Michael J. Fox Foundation for Parkinson’s Research. The primary goal of PPMI is to identify one or more biomarkers of Parkinson’s disease progression. This longitudinal study, following over 1000 subjects for up to 8 years, is taking place at 33 clinical sites in the United States, Europe, Israel and Australia. Subjects underwent clinical assessments, imaging and blood and CSF collection at predetermined time points. Further information can be found at http://www.ppmi-info.org.

### Standard protocol approvals, registrations, and patient consents

The PPMI study was approved by the Institutional Review Boards of each PPMI site. Informed written consent was obtained from all subjects at each site.

### Subject selection

In this study, we selected PD subjects who must meet clinical motor criteria for PD confirmed by dopamine transporter (DAT) imaging deficit with a diagnosis within 2 years and dementia-free based on the site investigator’s cognitive evaluation. Further information about the inclusion/exclusion criteria of PD adopted by the PPMI is described in detail at http://www.ppmi-info.org. Data were downloaded from http://www.ppmi-info.org on July 30, 2016 for this analysis. At the time of data acquisition, 423 PD subjects were enrolled, 362 PD subjects had completed 3 years of study follow-up. Eight PD subjects missed CSF data at baseline. Twelve PD subjects missed MoCA data at 3-year follow-up. A total of 350 PD subjects were included with completely clinical and neuropsychological assessments, and lumbar puncture at baseline, and 3 years follow-up visits for the neuropsychological assessments.

### RBD sleep assessments

We defined possible RBD individuals in PD with RBD sleep questionnaire (RBDSQ), in which a positive screen was defined as a score > 5 [17]. The RBD data used in this study were obtained from the PPMI files ‘REM_Sleep_Disorder_Questionnaire.csv’. According to this definition, the early PD subjects were allocated to PD with RBD and PD without RBD groups.

### Cognitive tests

The question 1 on part 1 of the Movement Disorder Society–Unified Parkinson’s Disease Rating Scale (MDS-UPDRS1.1) and Montreal Cognitive Assessment (MoCA) data sets used in this study were obtained from the PPMI files ‘MDS_UPDRS_Part_I.csv’ and ‘Montreal_Cognitive_Assessment__MoCA_.csv’ respectively. Question 1 on part 1 of the MDS-UPDRS was used to screen for PD reported cognitive impairment. MoCA is a validated global cognition test. The recommended cutoff score of < 26 was used to define mild cognitive impairment (MCI) [18]. Δ%MoCA over 3 years in each group were used to reflect cognitive decline and defined as:

$$ \Delta \%\mathrm{MoCA}=\left(\frac{\mathrm{MoCA}\ \mathrm{follow}-\mathrm{up}-\mathrm{MoCA}\ \mathrm{baseline}}{\mathrm{MoCA}\ \mathrm{baseline}}\right)\mathrm{x}\ 100 $$

In addition, individual cognitive tests for specific domains have been previously described [19] and included Verbal memory—the Hopkins Verbal Learning Test (HVLT) total recall, HVLT Recognition Discrimination, Visuospatial function—Benton Judgment of Line Orientation, Processing speed /attention—Symbol Digit Modalities Test, Executive function /working memory—Letter-Number Sequencing Test and Semantic Fluency. Mild cognitive impairment was defined as any 2 or more of the above cognitive tests > 1.5 SD below the standardized mean [19]. Δ% individual cognitive tests over 3 years in each group were used to reflect specific cognitive decline and defined as:

$$ \Delta \%\mathrm{cognitive}\ \mathrm{test}=\left(\frac{\mathrm{cognitive}\ \mathrm{test}\ \mathrm{follow}-\mathrm{up}-\mathrm{cognitive}\ \mathrm{test}\ \mathrm{baseline}}{\mathrm{cognitive}\ \mathrm{test}\ \mathrm{baseline}}\right)\mathrm{x}\ 100 $$

### Other clinical characteristics and assessments

The demographic, ApoE4 genotype, education and disease duration data were obtained from PPMI files. Hoehn and Yahr Stage was obtained to measure disease severity. Subscores of MDS-UPDRS part III were also collected at each visit to measure motor function. Assessments of NMS included (i) the University of Pennsylvania Smell Identification Test (UPSIT) with lower scores reflecting worse olfactory function, (ii) the State-Trait Anxiety Inventory (STAI), (iii) the 15-item Geriatric Depression Scale (GDS-15).

### CSF biomarkers

CSF Aβ_1–42_, alpha-synuclein, total tau (t-tau), and phosphorylated-tau at threonine 181 (p-tau) were measured by using Innogenetics (INNO-BIA AlzBio3) immunoassay kit–based reagents in the multiplex xMAPLuminex platform (Luminex). The CSF data used in this study were obtained from the PPMI files ‘Biospecimen_Analysis_Results.csv’. Further details of PPMI methods for CSF acquisition, and measurements and quality control procedures can be found at http://www.ppmi-info.org/.

### Statistical analysis

Baseline demographic, CSF biomarkers (Aβ_1–42_, t-tau, p-tau, alpha-synuclein), MoCA scores, individual cognitive tests, other motor and non-motor data were compared between two study groups using two-tailed Student *t* test for continuous variables and chi-square (χ2) tests for categorical variables, respectively. Normality assumptions were checked where appropriate. Kaplan-Meier survival analysis was used to estimate the effects of RBD on the progression to MCI. Seventy five PD subjects met criteria for MCI at baseline by MoCA criteria. Thus, PD subjects with baseline MCI were only excluded from Kaplan-Meier survival analysis.

We analyzed the association between all baseline CSF biomarkers and Δ% MoCA in each group. Linear regression models evaluated the predictive effects of all baseline CSF biomarkers on outcomes of Δ% MoCA over 3 years in each group. We further added an interaction term (CSF biomarkers*group) in the regression models to evaluate the interaction between CSF biomarkers and the 2 groups on the outcomes. Confounding variables based on biological rationality and published data were selected in linear regression model. Because UPDRS part III differed and Geriatric Depression Scale were borderline among the PD RBD and non-RBD subjects, linear regression model was corrected for UPDRS part III scores and Geriatric Depression Scale when analyzing the association between all baseline CSF biomarkers and Δ% MoCA. Age, gender, education, ApoE4 status were also included as covariates for analysis involving cognition. For each model, the coefficient (β) represents the difference in annual rate of change in the MoCA for each 1 point increase in the parameter (dependent variable) for the continuous variables. For the models involving categorical variables, each coefficient represents the difference in annual rate of change of the MoCA between the group. Statistical analysis were performed using SPSS (version 19.0) and a *P*-value < 0.05 was taken as statistically significant.

## Results

### Baseline demographic and clinical characteristics

Of the 350 early PD subjects analyzed, 136 were PD with RBD and 214 were PD without RBD. There were no differences in gender, age, education, ApoE4 genotype distribution, baseline disease duration, Hoehn and Yahr Stage, UPDRS1.1 positive cognition, MoCA scores and other NMS scores between two groups. There were significant differences detected in the baseline UPDRS part III in PD with RBD group compared to PD without RBD group (*P* = 0.044). The detailed characteristics of all these PD subjects were listed in Table 1.

**Table 1:** Baseline characteristics of PD stratified by RBD

	PD with RBD	PD without RBD	*P* value
	(*N* = 136)	(*N* = 214)	
Age, years	61.22 ± 9.44	60.42 ± 9.88	0.447
Males, n(%)	92(67.6%)	137(64.0%)	0.564
Education, year	15.61 ± 2.91	15.57 ± 2.97	0.890
ApoE4(%)	23.40%	24.50%	0.893
RBD	7.04 ± 1.91	2.53 ± 1.17	< 0.001*
Disease duration (months)	7.51 ± 6.69	7.35 ± 6.29	0.816
Hoehn and Yahr Stage	1.57 ± 0.49	1.51 ± 0.52	0.289
UPDRS part III	21.58 ± 9.41	19.63 ± 8.39	0.044*
UPDRS1.1 Cognition, N (% positive)	39(28.7%)	46(21.5%)	0.159
MoCA	26.98 ± 2.40	27.32 ± 2.21	0.170
UPSIT Total	21.60 ± 8.39	22.33 ± 7.96	0.411
Geriatric Depression Scale	5.39 ± 1.94	5.10 ± 1.37	0.065
Total STAI	93.58 ± 8.49	93.33 ± 7.58	0.771

Results are mean ± (SD). *P* value was assessed using two-tailed Student *t* test for each variable except gender, ApoE4 and UPDRS1.1, where chi-square (χ2) test was performed. *P value statistically significant. The age, education, ApoE4, disease duration, Hoehn and Yahr Stage, UPDRS1.1 Cognition, MoCA, UPSIT Total, depression and total STAI had no difference between two groups. However, the PD with RBD group had relative greater UPDRS part III

*Abbreviations: PD* Parkinsonr’s disease, *RBD* Rapid eye movement sleep behavioral disorder, *ApoE4* Apolipoprotein E ɛ4, *UPDRS* Unified Parkinson’s Disease Rating Scale, *MoCA* Montreal Cognitive Assessment, *UPSIT* University of Pennsylvania Smell Identification Test, *STAI* State Trait Anxiety Inventory

Baseline performance on the individual cognitive test among PD subjects with vs. without RBD are shown in Table 2. At baseline, those with RBD performed worse on the HVLT total recall (*P* = 0.039) and Symbol Digit Modalities (*P* = 0.008). Baseline Benton Judgment of Line Orientation and Letter-Number Sequencing tests also differed, however, they did not reach significance.

**Table 2:** Baseline specific cognitive characteristics of PD stratified by RBD

Cognitive domain	Measure	PD with RBD(*N* = 136)	PD without RBD(*N* = 214)	*P* value
Verbal memory	HVLT Total Recall	45.23 ± 10.80	47.69 ± 10.83	0.039*
	HVLT Recognition Discrimination	47.52 ± 13.05	49.70 ± 10.86	0.092
Visuospatial function	Benton Judgment of Line Orientation	12.65 ± 2.82	13.21 ± 2.58	0.058
Processing speed /attention	Symbol Digit Modalities	43.64 ± 9.08	46.36 ± 9.36	0.008*
Executive function /working memory	Letter-Number Sequencing	11.17 ± 2.53	11.72 ± 2.71	0.06
	Semantic Fluency total	50.19 ± 9.24	51.31 ± 10.40	0.305

Results are mean ± (SD). *P* value was assessed using two-tailed Student *t* test for each variable. *P value statistically significant. The PD with RBD group performed worse on the HVLT total recall and Symbol Digit Modalities

*Abbreviations: PD* Parkinsonr’s disease, *RBD* Rapid eye movement sleep behavioral disorder, *HVLT* Hopkins Verbal Learning Test

### Baseline CSF biomarkers

Baseline CSF biomarker levels by study groups were demonstrated in Table 3. There were no differences at baseline CSF t-tau, p-tau, alpha-synuclein, p-tau/t-tau ratio, and t-tau/Aβ_1–42_ ratio between two groups. CSF Aβ_1–42_ was lower in PD with RBD group compared to PD without RBD group (*P* = 0.005).

**Table 3:** Baseline CSF biomarkers of PD stratified by RBD

	PD with RBD	PD without RBD	*P* value
	(*N* = 136)	(*N* = 214)	
Aβ_1–42_ (pg/ml)	354.70 ± 103.45	384.73 ± 90.77	0.005*
t-tau (pg/ml)	43.76 ± 18.75	44.42 ± 16.89	0.736
p-tau (pg/ml)	14.57 ± 7.85	16.31 ± 11.32	0.121
alpha-synuclein (pg/ml)	1811.69 ± 741.35	1860.50 ± 818.55	0.578
p-tau/t-tau	0.35 ± 0.17	0.39 ± 0.25	0.130
t-tau/Aβ_1–42_	0.13 ± 0.07	0.12 ± 0.05	0.056

Results are mean ± (SD). *P* value was assessed using two-tailed Student *t* test for each variable. *P value statistically significant. The PD with RBD patients had significantly lower contents of CSF Aβ_1–42_

*Abbreviations: PD* Parkinsonr’s disease, *RBD* Rapid eye movement sleep behavioral disorder, *CSF* cerebrospinal fluid, *t-tau* total tau, *p-tau* phosphorylated-tau, *Aβ* β-amyloid

### Follow-up cognitive decline and conversion to MCI

**Fig. 1:**
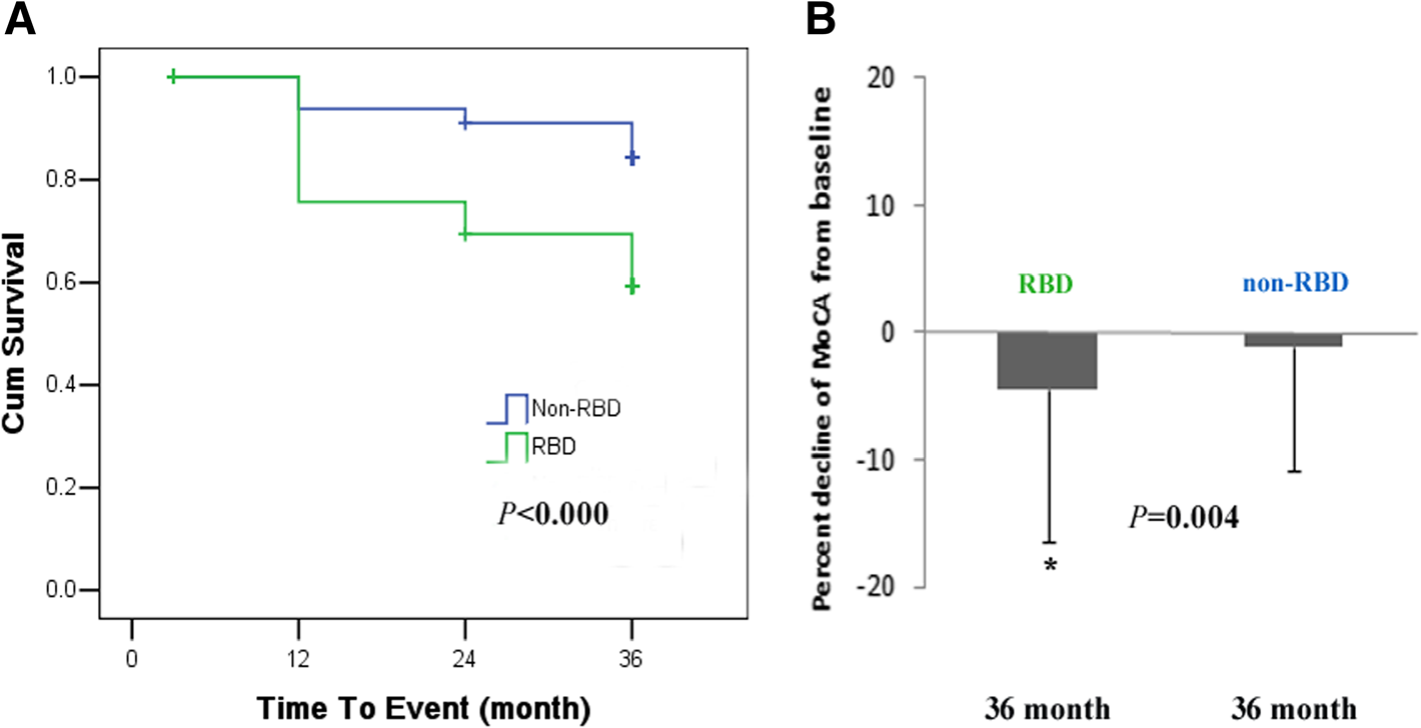
The effect of RBD on global cognitive decline in early PD subjects. **a** RBD predict conversion to MCI. Kaplan-Meier survival analysis was used to estimate the effects of RBD on the progression to MCI. **b** Cognitive decline by Δ%MoCA from baseline to 36 month in PD with RBD and without RBD. Results are mean ± (SD). *P* value was assessed using two-tailed Student *t* test for each variable. *P value statistically significant. Abbreviations: RBD, Rapid eye movement sleep behavioral disorder

On 3 year follow-up visits, those in PD with RBD group were more likely to report cognitive impairment (UPDRS1.1) compared to PD without RBD group (52.9% versus 31.8%, *P* < 0.001). The Δ% MoCA of PD subjects with RBD declined more than in those without RBD (*P* = 0.004). In Kaplan-Meier survival analysis with time to conversion to MCI as the dependent variable, there was a suggestion of an effect of RBD on the risk of developing MCI (*P* < 0.001, Fig. 1).

**Fig. 2:**
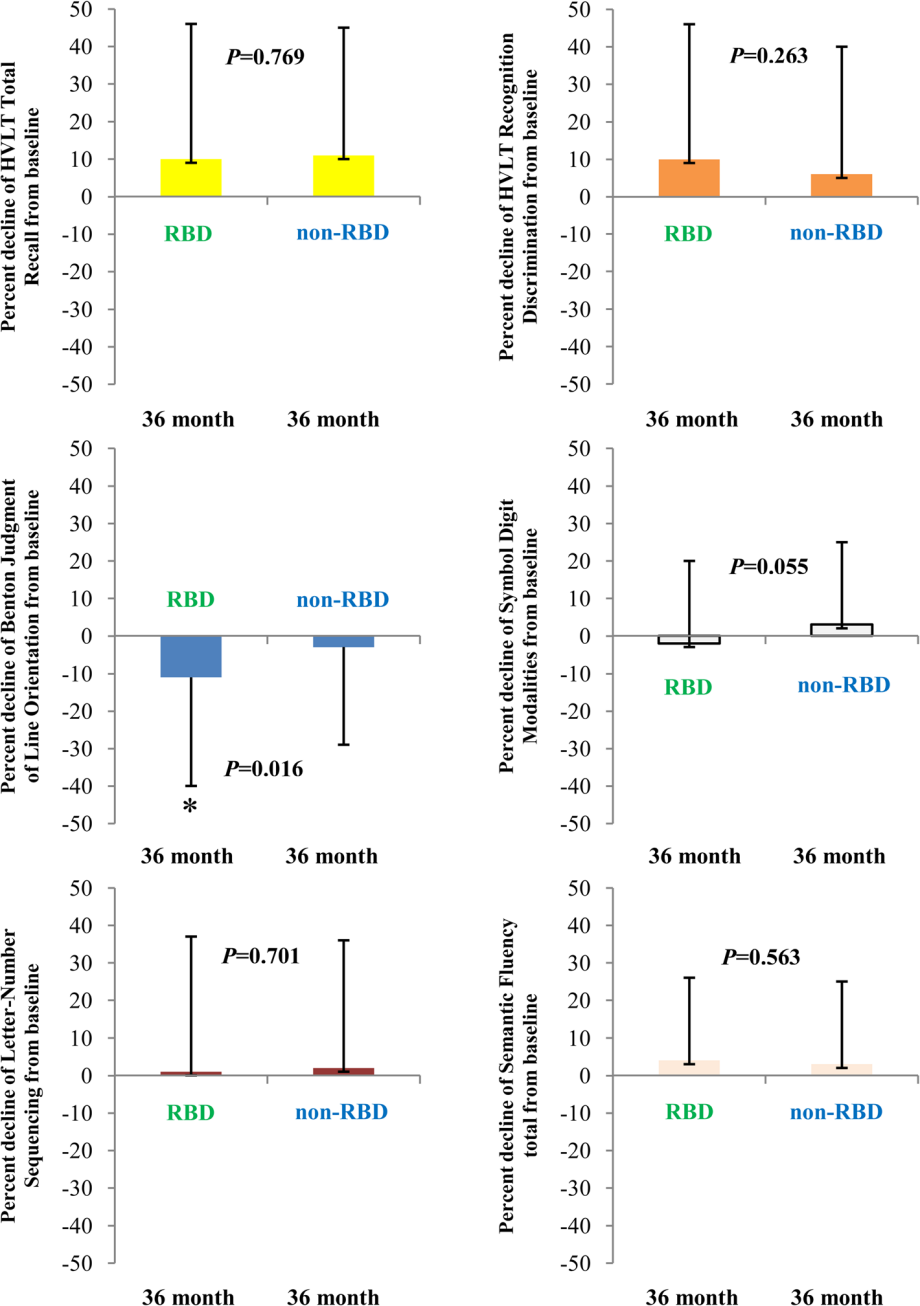
The effect of RBD on specific cognitive domains in early PD subjects. Cognitive decline by Δ%HVLT total recall, Δ%HVLT Recognition Discrimination, Δ% Benton Judgment of Line Orientation, Δ%Symbol Digit Modalities, Δ% Letter-Number Sequencing and Δ%Semantic Fluency from baseline to 36 month in PD with RBD and without RBD. Results are mean ± (SD). *P* value was assessed using two-tailed Student *t* test for each variable. *P value statistically significant. Abbreviations: RBD, Rapid eye movement sleep behavioral disorder; HVLT, Hopkins Verbal Learning Test

In a separate analysis, we defined MCI by individual cognitive tests criteria. We found that on 3 year follow-up visits, in visuospatial function domain, the Δ%Benton Judgment of Line Orientation of PD subjects with RBD declined more than in those without RBD (*P* = 0.016, Fig. 2). Similarly, PD subjects with RBD presented more MCI prevalence in comparision with PD subjects without RBD, however, they did not reach significance (15.0% versus 8.5%, *P* = 0.071).

### Baseline CSF Aβ_1–42_ as a predictor of cognitive decline in PD subjects with RBD

We further found that baseline CSF Aβ_1–42_ predicted global cognitive decline (Δ%MoCA) over 3 years in PD with RBD group (β = − 0.03, *P* = 0.003), but not in the PD without RBD group. For instance, MoCA scores decreased by 0.03 points for every 1 point reduction in CSF Aβ_1–42_ value. For example, a subject with an CSF Aβ_1–42_ value of 354 pg/ml could be expected to experience an additional 2.7 point decline in MoCA score over 3 years compared to a subject with an CSF Aβ_1–42_ value of 384 pg/ml. In addition, a significant interaction between Aβ_1–42_ and the 2 groups (Aβ_1–42_*group) confirmed that this effect was indeed higher in PD subjects with RBD than PD subjects without RBD (β = − 2.85, *P* = 0.014, Table 4).

**Table 4:** CSF biomarkers and RBD as predictors of global cognitive decline in PD

Variables (Δ%MoCA as dependent variable)	β	*P* value
Aβ_1–42_ (RBD group)	−0.03 ± 0.01	0.003*
Aβ_1–42_ (non-RBD group)	−0.001 ± 0.007	0.944
Group (categorical RBD and non-RBD)	2.95 ± 1.18	0.013*
Aβ_1–42_*Group	−2.85 ± 1.15	0.014*

Data are shown as coefficient (β) ± (SE). In these linear effects models, Δ%MoCA is the dependent variable and Aβ_1–42_, group and Aβ_1–42_*Group are the independent variable, with age, sex, education, ApoE4 status, disease duration and baseline UPDRS part III score as co-variates. *P value statistically significant. Significant interactive effects were observed in Aβ_1–42_ and RBD group

*Abbreviations: PD* Parkinsonr’s disease, *RBD* Rapid eye movement sleep behavioral disorder, *CSF* cerebrospinal fluid, *MoCA* Montreal Cognitive Assessment, *Aβ* β-amyloid

## Discussion

Based on the present results from PPMI database, we report that RBD is an predictor of cognitive decline in early PD patients and CSF Aβ_1–42_ is associated with cognitive decline in early PD with RBD.

RBD has been reported to be present in 15–72% of PD patients and is associated with poor parkinsonian symptoms, higher daily levodopa dosage, and cognitive impairment in more advanced PD patients [4, 7–10, 20]. However, in the large-scale, untreated, imaging-confirmed early PD cohort, we found that multiple baseline NMS didn’t show worse presentation in PD patients with RBD compared with PD patients without RBD, and they also remain relatively stable over 3 years (data not shown). While global cognition deteriorates in 3 years follow-up period in PD patients with RBD. Thus, we confirmed that RBD was still associated with cognitive decline in the early stage of PD.

There are several hypothesis that might explain the association between RBD and cognitive decline in PD [21, 22]. This is the first study to our knowledge exploring the underlyling basis for the association between RBD and cognitive decline within the untreated, imaging-confirmed early PD cohort. The ApoE4 is an established risk factor for Alzheimer’s disease [23–25] and has also been proposed to be an important risk factor for cognitive impairment in PD [13, 14]. Yet, our results showed that in early PD there was no difference in frequency of ApoE4 occurrence between PD with RBD and PD without RBD. In addition, in ApoE4 positive and ApoE4 negative PD paients with RBD, there was no difference in MoCA score loss (7.48 ± 11.04 versus 3.57 ± 11.05, *P* = 0.098) and Benton Judgment of Line Orientation (18.01 ± 25.03 versus 8.46 ± 29.13, *P* = 0.101). Thus, our data suggest that RBD increases the risk and severity of cognitive impairement in early stage of PD and is independent of ApoE4 status. Recent study even pointed out that ApoE4 did not affect cognitive performance in PD patients [26, 27]. Thus, the presence of ApoE4 does not increase the risk of becoming MCI at least in the first 3 years of early dementia-free PD with RBD.

Alzheimer’s disease (AD) pathology has been linked to cognitive decline in PD. Pathologic accumulation of Aβ is associated with PD patients with dementia. Cortical Aβ seem to determine the rate to dementia [28, 29]. Up to one third of patients who develop PD dementia will also meet pathologic criteria for AD, involving β-amyloid plaques and tau-containing neurofibrillary tangles, which may have an additive effect with alpha-synuclein pathology to worsen prognosis. The phosphorylated-tau at threonine 181 is one more specific biomarker for AD and is related to neurofibrillary tangles [30, 31]. CSF biomarkers, such as increased tau and decreased Aβ_1–42_, can reflect the degenerative process and have been associated with cognitive impairment in PD in cross sectional studies [15, 16, 32]. Yet, the relationship between CSF tau or Aβ_1–42_ and RBD in predicting cognitive decline in early PD remains unknown. In the present study, early PD patients were dichotomized into PD with RBD and PD without RBD based on RBD SQ score > 5 [17]. We found that in comparison to PD without RBD, only CSF Aβ_1–42_ was obviously lower in PD with RBD. Regression analysis showed that baseline CSF Aβ_1–42_ could predict cognitive decline over 3 years only in PD subjects with RBD. Similar studies with a relatively small sample of PD subjects have found a relationship between CSF Aβ_1–42_ levels and cognitive status in PD patients [33, 34]. Lower Aβ_1–42_ levels in the spinal fluid may be related to sequestration of the peptide from CSF into amyloid plaques. Lower CSF Aβ_1–42_ levels reflect greater amyloid plaque burden in patients with PD at risk for cognitive decline [35]. Therefore, our data suggest that lower CSF Aβ_1–42_ could be the closely related biomarker in cognitive decline in early PD with RBD. The addition of CSF Aβ_1–42_ to RBD testing may increase the likelihood of identifying those at high risk for cognitive decline in early PD.

Lewy body-type synucleinopathy is the main pathological marker in PD. PD dementia has also two major pathologic subgroups: neocortical synucleinopathy and neocortical synucleinopathy with Aβ deposition [28, 29]. Interestingly, there is inconsistent data about the relationship between CSF alpha-synuclein and cognitive impairment in PD patients. Several research found that high CSF alpha-synuclein correlated with PD dementia [36, 37] and low CSF total-α-synuclein was associated with dysfunction in phonetic-fluency (a frontal-lobe function) and with frontal cortical thinning in RBD [36]. Unlike several previous research, we did not show an association between CSF alpha-synuclein level and cognitive impairment in early PD. For high CSF alpha-synuclein, it is important to note that these previous research have focused on PD patients with dementia [36, 37], and the PPMI sample is relatively young and dementia-free. Our findings were consistent with some previous studies showing no association between CSF alpha-synuclein and cognitive impairment in early PD [32, 38]. Perhaps high CSF alpha-synuclein was more prevalent in PD patients with dementia. Our data supports the conclusion that CSF alpha-synuclein does not increase the risk of becoming MCI at least in the first 3 years of early dementia-free PD with RBD. Longer follow-up duration of PPMI cohort will reveal whether CSF alpha-synuclein is important in more advanced cognitive dysfunction of PD.

There are other baseline factors, including severity of disease, disease duration, education level, male gender, advanced age of onset, and concomitant medication which may be associated with more rapid cognitive decline in PD [39, 40]. However, in the early PD subjects from PPMI cohort, no significant differences were found in Hoehn and Yahr Stage, disease duration, onset age, education level, and male gender. There are few SSRI or SNRI usages in PD subjects in PPMI database. Previous study from PPMI database also demonstrated that the majority of PD patients with clinically significant depression were not treated with antidepressants [11]. So, these confounding factors were actually excluded when we investigated the relationship between CSF Aβ_1–42_ and cognitive decline in early PD with RBD. Meanwhile, there are very few diabetes or metabolic syndrome in these early PD subjects in PPMI cohort, so contribution of diabetes or metabolic syndrome to our findings is likely minimal. Additionally, although GBA mutations were more common in RBD, there are few GBA mutations carriers in PD subjects in PPMI database. Of the 350 early PD subjects analyzed, we identified only 6 PD with p.N370S GBA mutations. The confounding effect of p.N370S GBA mutations to our findings was likely minor.

Of course, we recognize that the present study has several limitations. First, the MoCA is one broadly-used and abbreviated instrument for global cognition assessment. The recommended cutoff scores < 26 was used to determine MCI (PD-MCI level I category). Since comprehensive testing may not always be available, an MCI definition based on an abbreviated assessment is very useful in routine clinical practice. The use of the MoCA alone is one level I category for defining PD-MCI by MDS guidelines and a diagnosis by this criteria is regarded less certain than a diagnosis of PD-MCI by level II category assessment. However, Analysis by MCI level II category assessment based on neuropsychological testing for specific domains (PD-MCI level II) did not reach significance likely due to low progression to MCI event rate as described in the present and previous research [41]. Second, the diagnosis of RBD was only based on clinical interview without sleep laboratory recording [17]. Thus, the diagnosis of RBD might not be formally confirmed. The onset time of RBD with PD was unknown in the PPMI cohort. There is no available data of RBD duration for PD subjects and the present analysis. Future study is still highly desirable in order to is disclose the more possible mechanism of RBD on cognition impairment of PD patients. Third, the neuropathological and neuroimaging correlates of lower CSF Aβ_1–42_ are not clear, which just can reflect the evidences for degeneration [42, 43]. In addition, cholinergic dysfunction is also associated with PD with RBD. However, longitudinal studies on large cohorts are required to gain more insight into the role of cholinergic dysfunction in the emergence of cognition impairment of PD patients with RBD [21]. Further studies based on this preliminary study are still required so as to contribute to a deeper understanding of cognitive impairement in PD with RBD, therapy planning and prognosis prediction for early PD patients with RBD.

## Conclusions

Taken together, this study provides evidence that CSF Aβ_1–42_ may play a role in prediction of cognitive impairment among early PD patients with RBD. As single marker, RBD may lack enough specificity. Early testing of CSF Aβ_1–42_ in such PD patients is important for providing more potential diagnostic value and determining next therapies.

## Abbreviations

ApoE4: Apolipoprotein E ɛ4
Aβ: β-amyloid
CSF: cerebrospinal fluid
HVLT: Hopkins verbal learning test
MoCA: Montreal cognitive assessment
PD: Parkinsonr’s disease
RBD: Rapid eye movement sleep behavioral disorder
STAI: State Trait Anxiety Inventory
UPDRS: Unified Parkinson’s Disease Rating Scale
UPSIT: University of Pennsylvania Smell Identification Test
